# Cancer risk perception among family members of patients with digestive tract cancer and associated patient- and family member-related factors: A cross-sectional study of 414 patient–family member pairs

**DOI:** 10.1016/j.apjon.2026.101026

**Published:** 2026-09-10

**Authors:** Luling Zhou, Lingzhi Yang, Lang Wang, Jiao Zhou, Ka Li

**Affiliations:** a West China School of Nursing, Sichuan University/West China Hospital, Sichuan University, Chengdu, Sichuan Province, China; b Chengdu BOE Hospital, Chengdu, Sichuan Province, China; c Medicine and Engineering Interdisciplinary Research Laboratory of Nursing & Materials, West China Hospital, Sichuan University/West China School of Nursing, Sichuan University, Chengdu, Sichuan Province, China

**Keywords:** Digestive tract cancer, Risk perception, Family member, Cross-sectional, Associated factors

## Abstract

**Objective:**

Family members of patients with digestive tract cancer may share familial or lifestyle-related risks, but their cancer risk perception is poorly understood. This study assessed cancer risk perception and associated patient- and family member-related factors.

**Methods:**

This cross-sectional study included 414 patient–family member pairs (828 individuals) from two tertiary hospitals in Sichuan Province, China, between March and October 2023. Cancer risk perception was assessed using a modified 26-item scale. Associated factors were examined using *t* tests, one-way analysis of variance, and hierarchical linear regression.

**Results:**

The mean total score among family members was 89.23 (standard deviation [SD] 22.42). Mean item scores were 3.41 (SD 0.99) for deliberative, 3.89 (SD 1.49) for affective, 3.76 (SD 1.03) for experiential, and 2.86 (SD 0.77) for carcinogenic factor risk perception. In the final model, patient treatment complications and mild self-care dependence were associated with higher scores. Among family members, higher scores were associated with junior college or higher education, monthly per capita family income greater than 5000 Chinese yuan (CNY), and previous care of another relative with cancer; lower scores were associated with older age (45–59 and ≥ 60 years), good self-rated health, and improved health status. The model explained 60.5% of the variance (adjusted *R*^*2*^ = 0.569; Δ*R*^*2*^ = 0.480).

**Conclusions:**

Cancer risk perception was associated with patient clinical circumstances and family member demographic, caregiving, and health characteristics. The carcinogenic factor dimension had the lowest mean item score. These findings support appropriately calibrated risk communication and guideline-based prevention counseling.

## Introduction

Cancer represents a major global health challenge and a leading cause of mortality. Among all malignancies, digestive tract cancers account for a substantial proportion of cancer incidence and deaths worldwide.^1^ In China, the burden of digestive tract cancers continues to escalate and imposes a particularly substantial economic strain due to the large population base.^2^, ^3^, ^4^ More importantly, these cancers often develop insidiously and present with nonspecific early clinical manifestations. Compounded by widespread cancer avoidance tendencies among the Chinese population, some patients may be reluctant to seek medical attention, thereby increasing the likelihood of advanced-stage presentation.^5^ Therefore, beyond treatment, identifying risk populations and promoting timely preventive actions are essential for digestive tract cancer control.

Within this prevention framework, family members of patients with digestive tract cancer deserve particular attention. Digestive tract cancers often exhibit familial aggregation, and family history has been associated with an increased risk of digestive tract cancers, particularly among first-degree relatives.^6^^,^^7^ This elevated risk may arise from the combined effects of genetic susceptibility, shared household environments, similar dietary and lifestyle habits.^8^ Furthermore, traditional Chinese dining customs and frequent close contact may facilitate the transmission of *Helicobacter pylori*, a recognized risk factor for gastric cancer.^9^ Family members not only endure caregiving burdens and financial strain, but also confront psychological distress due to the patient's condition and potential bereavement. The accumulation of multiple high-risk factors may prompt family members to adopt unhealthy coping behaviors.^10^ Therefore, family members are not only caregivers, but also individuals who may require targeted prevention guidance because of their own potential cancer risk.

Risk perception provides a key conceptual link between elevated objective risk and preventive action. According to the Health Belief Model, perceived susceptibility and perceived severity are important determinants of whether individuals adopt preventive health behaviors. For family members of patients with digestive tract cancer, appropriate risk perception may help them pay attention to modifiable risk factors and make informed screening decisions when they meet relevant eligibility criteria. In contrast, underestimation or overestimation of risk perception may lead to neglect of prevention or unnecessary cancer worry. In this study, risk perception refers to family members' subjective understanding and emotional response regarding their likelihood of developing digestive tract cancer. Rather than being limited to a rational estimation of probability, risk perception includes multiple components. The tripartite model of risk perception (TRIRISK) conceptualizes it as deliberative, affective, and experiential dimensions, reflecting analytical judgment, emotional concern, and intuitive feelings, respectively.^11^ This framework is particularly suitable for family members of patients with cancer, because their perceptions may be shaped by knowledge, family history, caregiving experience, observation of the patient's illness trajectory, and psychological distress.^12^

The assessment and guidance of family members' risk perception are highly relevant to oncology nursing practice. During hospitalization, treatment, and follow-up, family members are repeatedly involved in symptom observation, dietary management, treatment decision-making, psychological support, and post-discharge care. These interactions create continuous opportunities for nurses to assess family members' perceived risk, identify misconceptions, and provide individualized education on modifiable carcinogenic factors. Integrating risk perception into family-centered cancer care may help nurses support both the patient's treatment process and the family member's own preventive health behaviors.

Previous studies among family members of patients with digestive tract cancer mainly focused on family member-related variables, such as age, educational level, cancer worry, health belief, lifestyle, and screening behaviors.^10^^,^^13^, ^14^, ^15^ Patient-level clinical information, such as treatment complications or self-care ability, has rarely been incorporated into the analysis. However, family members' perceived cancer risk may be influenced not only by their own demographic, health, and caregiving characteristics but also by the patient's disease context. Therefore, this study aimed to assess cancer risk perception among family members of patients with digestive tract cancer and to identify its associated factors from the perspectives of both patients and family members, thereby providing evidence for family-centered risk communication and oncology nursing practice.

## Methods

### Study design and samples

In this study, digestive tract cancers refer specifically to esophageal, gastric, and colorectal cancers. These three cancers were grouped together because they arise from the continuous gastrointestinal tract and are commonly classified as major digestive system cancers. In addition, these anatomical sites directly contact ingested food and its metabolites, and are therefore exposed to partly overlapping dietary factors, food-borne carcinogens, and microbial environments. This common exposure pathway provides a biological basis for shared carcinogenic mechanisms across these cancers. More importantly, esophageal, gastric, and colorectal cancers share several public health and prevention-related characteristics, including familial aggregation, lifestyle-related risk factors, potential influence of shared household environments, and established opportunities for early detection and screening.

This cross-sectional study employed a sequential inclusion method to recruit participants from two tertiary hospitals in Sichuan Province, China, between March and October 2023. This manuscript represents a secondary analysis based on the same dataset as our previously published study on disease self-monitoring behaviors among family members of patients with digestive tract cancer.^16^ This study used paired patient–family member recruitment and linked data, but was not a formal dyadic study. Patients and their family members were recruited in 1:1 pairs. Eligible patients were required to: 1) have a confirmed diagnosis of esophageal, gastric, or colorectal cancer; 2) be receiving cancer-related treatment during the study period, including surgery, chemotherapy, radiotherapy, targeted therapy, immunotherapy, or other standard anticancer treatments; 3) be able to understand the questionnaire and provide informed consent; and 4) have at least one eligible family member willing to participate. Family members were eligible if they met the following criteria: 1) ≥ 18 years old; 2) awareness of the patient's diagnosis; 3) be spouses or biological relatives of the patient, including first-degree and other blood relatives; 4) willing to participate. Exclusion criteria were: 1) have cognitive impairment, mental disorders, or communication difficulties; 2) a prior cancer diagnosis in the family member; 3) prior participation of another family member in the survey.

During recruitment, eligible patients were first identified according to the inclusion and exclusion criteria. Each patient was then invited to nominate one family member who met the eligibility criteria. When more than one eligible family member was available, the family member who was willing and available to participate was recruited. Each patient–family member pair was assigned a unique identification code to ensure accurate linkage of paired data. The rationale for this pairing was to link family members' cancer risk perception with the corresponding patient's disease-related and family-context information. Therefore, the 1:1 pairing was not intended to create matched comparison groups or to control for confounding through matching variables.

The sample size was estimated using the standard formula for cross-sectional studies: $N={\left(\frac{Z_{1-\alpha /2}\sigma }{\delta }\right)}^{2}$. Based on a pre-survey, the standard deviation (σ) of cancer risk perception score was 22.18. With α set at 0.05 ($Z_{1-\alpha /2}$ = 1.96) and a margin of error (δ) of 2.5, the minimum required sample size was 302 patient–family member pairs, corresponding to 302 family member outcome observations. Ultimately, the study included 414 patient–family member pairs, representing 828 individual participants.

### Measurements

#### Demographic information of patients

This questionnaire was self-designed based on previous studies and in accordance with the research objectives. It included social demographic characteristics and disease-related information. The social demographic information consisted of sex, age, educational level, and family per capita monthly income. The disease-related information included 7 items: type of digestive tract cancer, clinical stage, underwent surgery, treatment for cancer recurrence, treatment complications, comorbidities, and self-care ability. Clinical stage was determined according to the tumor–node–metastasis (TNM) staging information recorded in the medical records. For analysis, early stage, corresponding to TNM stage 0/carcinoma in situ and stage I; middle stage, corresponding to TNM stage II–III, including locally advanced disease or regional lymph node involvement without distant metastasis; and late stage, corresponding to TNM stage IV, indicating distant metastasis. Treatment complications were defined as adverse reactions or complications related to cancer treatment, including myelosuppression, skin reactions, gastrointestinal reactions, etc. These data were derived from the medical records and patient self-reports, covering the period from the initiation of cancer treatment to questionnaire completion. Self-care ability was evaluated using the Barthel Index, including eating, bathing, grooming, dressing, control urination and defecation, using the toilet, transferring bed and chair, walking on flat ground, and ascending/descending stairs. The total score ranges from 0 to 100, with higher scores indicating greater independence. In this study, self-care ability was classified as no dependence required (100 points), mild dependence (61–99 points), moderate dependence (41–60 points), and severe dependence (≤ 40 points).

#### Demographic information of family members

This questionnaire was also independently compiled by the researchers, consisting of three parts: socio-demographic characteristics of family members, caring experience, and their own health conditions. Socio-demographic information encompassed 11 items, including sex, age, educational level, religious belief, employment and marital status, family per capita monthly income, first-degree relatives of the patient, situation of living with patient, place of residence, and medical insurance. The care experience covered participating in patient care, communicating with the doctor about patient's condition, other relatives who had cancer, participating in the care of other relatives with cancer, and experienced cancer bereavement. The health condition was assessed through diagnosis of related diseases, self-rated health, and change in health status compared to the state before the patient's cancer diagnosis.

#### Cancer risk perception scale

Cancer Risk Perception Scale for digestive tract cancers consisted of the Chinese version of The Tripartite Model of Risk Perception, TRIRISK^17^ and the perception of digestive tract cancer risk factors. TRIRISK was developed by Ferrer,^11^ based on literature review and expert group discussion, and was used to assess the public perceptions of personal susceptibility to chronic diseases. The scale was subsequently translated into Chinese and validated, with psychometric analyses confirming its satisfactory reliability and validity.^17^

In June 2022, given that the original scale assessed general chronic disease risk perception without addressing disease-specific risk factors, we further added a dimension assessing perceived risk related to major risk factors for digestive tract cancers. Other risk-perception instruments also include content related to perceived risk factors, which further highlights the need to include this dimension.^18^, ^19^, ^20^ The initial item pool for this additional dimension was generated through a review of authoritative evidence on risk factors for esophageal, gastric, and colorectal cancers. The main sources included reviews and epidemiological studies.^21^, ^22^, ^23^ Based on these sources, initial items covered major risk factors such as unhealthy dietary habits, insufficient intake of vegetables and fruits, high intake of processed or preserved foods, tobacco smoking, alcohol consumption, physical inactivity, overweight or obesity, family history of cancer, and previous digestive tract diseases or precancerous lesions.

The research team then screened the items according to the following principles: (1) the factor was supported by previous evidence or guidelines as being related to esophageal, gastric, or colorectal cancer; (2) the item was understandable to non-medical respondents; (3) the item reflected perceived cancer risk rather than factual knowledge or actual behavior; (4) the item was applicable to family members of patients with digestive tract cancers; and (5) items with overlapping meanings were merged or deleted. After discussion, a preliminary version of the additional dimension was formed.

Expert consultation was conducted to further evaluate the relevance and clarity of the newly developed items. Experts from oncology nursing, gastrointestinal oncology, and instrument development were invited to assess each item in terms of relevance to digestive tract cancer risk perception, scientific accuracy, and applicability to the target population. The item relevance was scored using the Likert five-point scale. A total of nine experts were invited to participate in the cultural adaptation and expert consultation of the scale. Items with low relevance and unclear wording were revised or deleted according to expert feedback. The expert authority coefficient (Cr) reached 0.89. The S-Content Validity Index (scale-level CVI) of Cancer Risk Perception Scale was 0.88. Except for the item-level CVI (I-CVI) of item two which was 0.67, the I-CVI of the other items ranged from 0.78 to 1.00. Some experts had suggested that item two was similar to item 1. After discussion by the research team, item two was retained because these two items evaluated risk perception from both qualitative and quantitative perspectives. And this item was not a newly added item in this study. It can also be consistent with the original scale.

Before formal data collection, a pilot test was conducted among 30 eligible family members to evaluate the clarity, comprehensibility, and feasibility of the questionnaire. These 30 family members were recruited separately. Participants demonstrated clear comprehension of all scale items and completed the questionnaire without difficulty.

An unpublished supplementary exploratory factor analysis of the modified 26-item scale was conducted using data from 200 participants selected from the earlier scale-validation dataset between October and December 2022. These 200 participants were selected because they had completed all 26 items, including the 18 Chinese TRIRISK items and the eight newly added carcinogenic factor risk perception items, during the earlier stage. These 200 participants were not included in the 414 participants used for the present analyses. All participants in the previous scale-validation study were independent of the 414 family members included in the present study. In this analysis, the Kaiser–Meyer–Olkin (KMO) was 0.901, and Bartlett's test of sphericity was significant (*P* < 0.001), indicating that the data were suitable for factor analysis. A total of four factors were extracted, explaining 69.48% of the total variance. The factor loadings of the retained items ranged from 0.496 to 0.935, supporting the preliminary psychometric evidence. The final scale consisted of 26 items across four dimensions: deliberative risk perceptions (6 items), affective risk perceptions (6 items), experiential risk perceptions (6 items), and carcinogenic factor risk perception (8 items). Except for item two which was measured by percentages, the rest of the items were rated on a Likert seven-point scale. The item two needed to be converted into a 7-point scale using a formula: percentage score / 100% = conversion score / 7. The scores were rounded off, and then all the items were summed up to calculate the total score. Item 16 was reverse-scored. Higher scores indicated greater perceived personal risk of developing digestive tract cancer.

In the current sample of 414 family members, the modified 26-item scale showed good internal consistency. The Cronbach's α coefficient was 0.946 for the full scale, 0.933 for deliberative risk perception, 0.977 for affective risk perception, 0.902 for experiential risk perception, and 0.729 for carcinogenic factor risk perception.

### Data collection

To ensure the accuracy and completeness of the data, all data collection was carried out by the same researcher. The researcher conducted a detailed training on the questionnaire collection and the survey content before the collection process. After identifying eligible patients and family members, the researcher provided a detailed explanation of the study's purpose, significance, and relevant precautions, then secured informed consent. Electronic self-administered questionnaires were used when participants were able and willing to complete the questionnaire independently using a smartphone or other electronic device. Face-to-face interviews were used when participants had difficulty using electronic devices, had limited reading ability, or preferred interviewer assistance. The questionnaire content, item order, and response options were identical across both modes. During interviews, the researcher read each item and response option neutrally without explanation, guidance, or inducement. To ensure data quality, each questionnaire was checked immediately after completion by the researcher. After data collection was completed, the supervisor reviewed the original data to further verify data completeness and accuracy.

Patients and family members completed their questionnaires independently. Cancer Risk Perception Scale for digestive tract cancers was filled out solely by family members. Patients did not complete this scale. Patient-related information was collected only to examine potential patient-related factors associated with family members’ risk perception. The questionnaire collection process typically required 15–25 minutes per participant. A total of 436 patient–family member pairs were invited to participate. Among them, 414 pairs, representing 828 individual participants, provided complete and valid paired data and were included in the final analysis, yielding a valid pair-level response rate of 94.95%. The remaining 22 pairs were excluded because the family members requested to withdraw before completing the questionnaire. There were no missing values in the valid questionnaires.

### Ethical consideration

Before data collection, both patients and their family members were informed of the study purpose, procedures, voluntary nature of participation, confidentiality measures, and potential risks and benefits. Written informed consent was obtained independently from each patient and each family member. For participants who completed the electronic questionnaire, informed consent was obtained electronically before they accessed the questionnaire items. Participants were informed that they could withdraw from the study at any time without any negative effect on their medical care or relationship with the research team. All questionnaire data were coded and used only for research purposes. The data were stored securely, with access limited to authorized members of the research team. This study was approved by the Biomedical Ethics Committee of West China Hospital, Sichuan University (Approval No. 2022–1261), and the approval covered both participating hospitals. The parent observational study was prospectively registered in the Chinese Clinical Trial Registry (ChiCTR2200066442).

### Data analysis

Statistical analyses were performed using SPSS 25.0. Categorical data were expressed as percentages. Normality test of the digestive tract cancer risk perception scale scores was assessed using the Shapiro–Wilk test and visual inspection of histograms. Data that were approximately normally distributed were described statistically using the mean ± SD. Homogeneity of variance was assessed using Levene's test. For normally distributed variables with homogeneous variance, independent-samples *t*-tests or one-way ANOVA were used; otherwise, Mann–Whitney U tests or Kruskal–Wallis tests were applied.

Variables with *P* < 0.05 in univariate analyses and variables considered clinically or theoretically important were entered into hierarchical linear regression analysis. Patient-related variables were entered in Model 1, including patient education level, underwent surgery, treatment complications, and self-care ability. Family member-related variables were added in Model 2, including age, education level, employment status, religious belief, family per capita monthly income, first–degree relationship with patient, situation of living with patient, place of residence, medical insurance, communicating with the doctor about patient's condition, other relatives who had cancer, participating in the care of other relatives with cancer, experience of cancer bereavement, diagnosis of related diseases, self-rated health, and change in health status. For each multilevel categorical variable, dummy variables were created with one category specified as the reference category. In addition to the category-specific coefficients, an overall test was performed for each multilevel categorical factor. Model fit and diagnostics were assessed using *R*^*2*^, adjusted, Δ*R^2^* *F* test, *P* value, overall *P* value, and variance inflation factors. The relevant residual and influence diagnostics included examination of residual plots, standardized residuals, leverage values, and Cook's distance. A two-tailed *P* < 0.05 was considered statistically significant.

## Results

### Demographic characteristics

This study recruited 414 patient–family member pairs, representing 414 patients with digestive tract cancer and 414 family members. The grouping criteria for general characteristics were determined with reference to previous literature, commonly used demographic classification standards, local socioeconomic conditions, and the actual distribution of the sample.

Among the patients, 291 were male (70.3%); their ages ranged from 24 to 90 years old, among which 180 (43.5%) were between 60 and 74 years old; and 120 (29.0%) had an educational level of primary school or below. The demographic characteristics and disease conditions of the patients are detailed in Table 1.

**Table 1:** Demographic and clinical characteristics of patients (*N* = 414).

Variable	*n* (%)	Variable	*n* (%)
**Age (years)**		**Clinical staging of cancer**	
≤ 44	31 (7.5)	Early stage	52 (12.5)
45–59	173 (41.8)	Middle stage	206 (49.8)
60–74	180 (43.5)	Late stage	156 (37.7)
≥ 75	30 (7.2)	**Underwent surgery**	
**Sex**		Yes	239 (57.7)
Male	291 (70.3)	No	175 (42.3)
Female	123 (29.7)	**Treatment for cancer recurrence**	
**Education level**		Yes	77 (18.6)
Primary school and below	120 (29.0)	No	337 (81.4)
Middle school	118 (28.5)	**Treatment complications**	
High school level or above	176 (42.5)	Yes	147 (35.5)
**Family per capita monthly income(CNY)**		No	267 (64.5)
≤ 2000	112 (27.1)	**Comorbidities**	
2001–5000	178 (43.0)	Yes	336 (81.2)
> 5000	124 (29.9)	No	78 (18.8)
**Type of cancer**		**Self-care ability**	
Esophageal cancer	113 (27.3)	No dependence required	149 (36.0)
Gastric cancer	153 (37.0)	Mild dependence	199 (48.0)
Colorectal cancer	148 (35.7)	Moderate dependence	55 (13.3)
		Severe dependence	11 (2.7)

In terms of family members, there were 257 females (62.1%), with ages ranging from 19 to 78 years. A total of 178 participants (43.0%) were 44 years or younger. 241 (58.2%) were employed and 195 (47.1%) were first-degree relatives of the patient. The demographic data, care experiences, and health conditions of the family members are presented in Table 2.

**Table 2:** Demographic, caregiving and health characteristics of family members (*N* = 414).

Variable	*n* (%)	Variable	*n* (%)
**Age (years)**		**Place of residence**	
≤ 44	178 (43.0)	Rural area	82 (19.8)
45–59	164 (39.6)	Suburb area	97 (23.4)
≥ 60	72 (17.4)	Urban area	235 (56.8)
**Sex**		**Medical insurance**	
Male	157 (37.9)	Basic medical insurance	317 (76.6)
Female	257 (62.1)	Supplementary insurance	92 (22.2)
**Education level**		Self-funded	5 (1.2)
Primary school and below	145 (35.0)	**Participating in patient care**	
Middle school	73 (17.6)	Never	5 (1.2)
Junior college	84 (20.3)	Occasionally	52 (12.6)
Bachelor's degree or above	112 (27.1)	Sometimes	116 (28.0)
**Employment status**		Often	180 (43.5)
Farmer	12 (2.9)	Always	61 (14.7)
Retired	81 (19.6)	**Communicating with the doctor about patient's condition**	
Employed	241 (58.2)	Yes	342 (82.6)
Unemployed	80 (19.3)	No	72 (17.4)
**Religious belief**		**Other relatives who had cancer**	
Yes	16 (3.9)	Yes	84 (20.3)
No	398 (96.1)	No	330 (79.7)
**Family per capita monthly income(CNY)**		**Participating in the care of other relatives with cancer**	
≤ 2000	54 (13.0)	Yes	24 (5.8)
2001–5000	152 (36.7)	No	390 (94.2)
> 5000	208 (50.3)	**Experienced cancer bereavement**	
**First-degree relatives of the patient**		Yes	68 (16.4)
Yes	195 (47.1)	No	346 (83.6)
No	219 (52.9)	**Diagnosis of related diseases**	
**Marital status**		Yes	120 (29.0)
Married	380 (91.8)	No	294 (71.0)
Single/divorced/widowed	34 (8.2)	**Self-rated health**	
**Situation of living with patient**		Poor	42 (10.1)
Not living with the patient	141 (34.1)	Average	190 (45.9)
Living with the patient all the time	226 (54.6)	Good	182 (44.0)
Living intermittently with the patient	47 (11.3)	**Change in health status**	
		Worse	89 (21.5)
		No change	302 (72.9)
		Improved	23 (5.6)

Diagnosis of related diseases was defined as any current or previous physician-diagnosed diseases reported by family members, regardless of disease type.

### The score results of cancer risk perception scale

The scale scores followed a normal distribution. The total score of cancer risk perception scale among family members was 89.23 ± 22.42 (range: 39–138), with an average item score of 3.43 ± 0.86. The average item score of each dimension was as follows: deliberative risk perception (3.41 ± 0.99), affective risk perception (3.89 ± 1.49), experiential risk perception (3.76 ± 1.03), and carcinogenic factor risk perception (2.86 ± 0.77).

### Differences in cancer risk perception by patient and family member characteristics

The Levene's test for variance homogeneity showed that *P* values were all greater than 0.05, indicating that the variances were homogeneous. The results of the univariate analysis showed that the patient's educational level, family per capita monthly income, underwent surgery, treatment complications, and self-care ability were associated factors of the family members' perception of cancer risk (Table 3). The family members' own age, educational level, employment status, religious belief, being the first-degree relative of the patient, place of residence, living with the patient, family per capita monthly income, medical insurance, communicating with the doctor about the patient's condition, caregiving experience, and their own health status were associated factors of the family members' perception of cancer risk (Table 4).

**Table 3:** Univariate analyses of patient characteristics and cancer risk perception.

Characteristics	Score	*t/F*	*P* value	Characteristics	Score	*t/F*	*P* value
**Age (years)**		0.238	0.870	**Clinical staging of cancer**		1.248	0.288
≤ 44	87.70±24.03			Early stage	90.03±20.40		
45–59	88.41±21.25			Middle stage	87.52±23.12		
60–74	90.11±23.18			Late stage	91.22±22.09		
≥ 75	90.25±23.58			**Underwent surgery**		2.763	0.006
**Sex**		−0.867	0.386	Yes	91.81±22.70		
Male	88.61±22.82			No	85.70±21.62		
Female	90.70±21.47			**Treatment complications**		3.862	<0.001
**Education level**		13.606	<0.001	Yes	94.87±21.24		
Primary school and below	83.84±23.61			No	86.12±22.49		
Middle School	85.06±21.65			**Treatment for cancer recurrence**		1.600	0.110
High school level or above	95.70±20.51			Yes	92.91±23.41		
**Family per capita monthly income (CNY)**		30.330	<0.001	No	88.39±22.14		
≤ 2000	80.81±21.77			**Comorbidities**		1.163	0.246
2001–5000	87.14±23.11			Yes	89.84±22.19		
> 5000	99.83±17.56			No	86.57±23.39		
**Type of cancer**		0.643	0.526	**Self-care ability**		2.719	0.044
Esophageal cancer	90.03±23.87			No dependence required	85.47±20.93		
Gastric cancer	87.60±21.21			Mild dependence	91.16±23.64		
Colorectal cancer	90.30±22.57			Moderate dependence	93.39±21.39		
				Severe dependence	84.45±18.68

**Table 4:** Univariate analyses of family member characteristics and cancer risk perception.

Family characteristics	Score	*t/F*	*P* value	Family characteristics	Score	*t/F*	*P* value
**Age (years)**		20.256	<0.001	**Place of residence**		14.419	<0.001
≤ 44	96.54±21.71			Rural area	80.50±20.33		
45–59	85.62±21.53			Suburb area	84.86±22.88		
≥ 60	79.38±20.56			Urban area	94.08±21.74		
**Sex**		0.223	0.823	**Medical insurance**		10.711	<0.001
Male	89.54±21.93			Basic Medical Insurance	86.53±22.23		
Female	89.04±22.76			Supplementary insurance	98.54±20.75		
**Education level**		84.354	<0.001	Self-funded	88.54±22.79		
Primary school and below	73.95±17.62			**Participating in patient care**		0.406	0.802
Middle School	85.53±18.68			Never	88.38±35.95		
Junior College	95.35±21.10			Occasionally	88.45±18.36		
Bachelor's degree or above	106.82±15.93			Sometimes	91.03±22.99		
**Employment status**		26.303	<0.001	Often	87.83±23.19		
Farmer	65.42±14.19			Always	90.66±21.36		
Retired	87.89±19.34			**Communicating with the doctor about patient's condition**		5.376	<0.001
Employed	95.12±21.63			Yes	91.86±22.01		
Unemployed	76.40±20.98			No	76.73±20.17		
**Religious belief**		−2.604	0.010	**Other relatives who had cancer**		5.088	<0.001
Yes	75.01±22.07			Yes	100.02±21.14		
No	89.80±22.28			No	86.48±21.94		
**Family per capita monthly income (CNY)**		113.418	<0.001	**Participating in the care of other relatives with cancer**		−3.330	0.001
≤ 2000	69.32±16.84			Yes	103.85±19.08		
2001–5000	78.53±19.30			No	88.33±22.32		
> 5000	102.21±17.38			**Experienced cancer bereavement**		−4.832	<0.001
**First-degree relatives of the patient**		6.242	<0.001	Yes	100.93±21.91		
Yes	96.20±21.91			No	86.93±21.83		
No	83.02±21.05			**Diagnosis of related diseases**		2.488	0.013
**Marital status**		0.251	0.802	Yes	93.49±21.87		
Married	89.14±22.39			No	87.49±22.45		
Single/divorced/widowed	90.15±23.09			**Self-rated health**		56.422	<0.001
**Situation of living with patient**		10.017	<0.001	Poor	95.60±23.68		
Not living with the patient	93.93±21.16			Average	98.88±19.67		
Living with the patient all the time	84.86±22.01			Good	77.67±19.36		
Living intermittently with the patient	96.10±23.93			**Change in health status**		18.282	<0.001
				Worse	98.66±21.54		
				No change	87.91±21.43		
				Improved	69.97±23.06		

Patient-reported family per capita monthly income was significant in the univariate analysis. Given that its concept and statistics overlap with the household income reported by family members, it was not included in the hierarchical linear regression model. Cancer risk perception was reported by family members and family member-reported household income was considered more proximal to the outcome.

### Hierarchical linear regression analysis of cancer risk perception

The variable coding is shown in Table 5. Hierarchical linear regression analyses of cancer risk perception are presented in Table 6. Model 1, which included patient-related variables, was statistically significant and explained 12.5% of the variance in cancer risk perception (adjusted *R*^*2*^ = 0.110). After adding family member-related variables, Model two explained 60.5% of the variance in cancer risk perception (adjusted *R*^*2*^ = 0.569), representing an *R*^*2*^ increase of 0.480. In the final model, the associated factors of cancer risk perception were treatment complications, mild dependence in patients' self-care ability, age 45–59 years and ≥ 60 years of family members, junior college and bachelor's degree or above, family per capita monthly income > 5000 CNY, participation in caring for other relatives with cancer, good self-rated health, and improved health status.

**Table 5:** Independent variable assignment.

Variable		Assignment values
**Patient**	Education level	Primary school and below (0,0); middle school (1,0); high school level or above (0,1)
	Underwent surgery	Yes = 1; No = 0
	Treatment complications	Yes = 1; No = 0
	Self-care ability	No dependence required (0,0,0); mild dependence (1,0,0); moderate dependence (0,1,0); severe dependence (0,0,1)
**Family member**	Age (years)	≤ 44 (0,0); 45–59 (1,0); ≥ 60 (0,1)
	Education level	Primary school and below (0,0,0); middle school (1,0,0); junior college (0,1,0); Bachelor's degree or above (0,0,1)
	Employment status	Unemployed (0,0,0); Farmer (1,0,0); Retired (0,1,0); employed (0,0,1)
	Religious belief	Yes = 1; No = 0
	Family per capita monthly income (CPY)	≤ 2000 (0,0); 2001–5000 (1,0); > 5000 (0,1)
	First-degree relatives of the patient	Yes = 1; No = 0
	Situation of living with patient	Not living with the patient (0,0); living with the patient all the time (1,0); living intermittently with the patient (0,1)
	Place of residence	Rural area (0,0); Suburb area (1,0); Urban area (0,1)
	Medical insurance	Basic medical insurance (0,0); supplementary insurance (1,0); self-funded (0,1)
	Communicating with the doctor about patient's condition	Yes = 1; No = 0
	Other relatives who had cancer	Yes = 1; No = 0
	Participating in the care of other relatives with cancer	Yes = 1; No = 0
	Experienced cancer bereavement	Yes = 1; No = 0
	Diagnosis of related diseases	Yes = 1; No = 0
	Self-rated health	Poor (0,0); average (1,0); good (0,1)
	Change in health status	Worse (0,0); No change (1,0); improved (0,1)

**Table 6:** Hierarchical linear regression analysis of cancer risk perception (*N* = 414).

Variables			*B*	*SE*	*β*	*t*	*P* value	*Overall P* value	*95% CI*	*VIF*
**Model 1**	(Constant)		74.303	2.780		26.729	< 0.001		(68.838, 79.768)	
	Education level	Primary school and below	Ref					< 0.001		
		Middle school	1.119	2.820	0.023	0.397	0.692		(−4.425, 6.663)	1.499
		High school level or above	10.917	2.617	0.241	4.172	< 0.001		(5.773, 16.061)	1.548
	Underwent surgery	No	Ref					0.029		
		Yes	4.758	2.170	0.105	2.192	0.029		(0.491, 9.025)	1.063
	Treatment complications	No	Ref					< 0.001		
		Yes	7.815	2.199	0.167	3.553	< 0.001		(3.491, 12.139)	1.025
	Self-care ability	No dependence required	Ref					0.008		
		Mild dependence	7.192	2.332	0.160	3.084	0.002		(2.608, 11.777)	1.256
		Moderate dependence	7.823	3.359	0.119	2.329	0.020		(1.221, 14.425)	1.202
		Severe dependence	−2.027	6.643	−0.015	−0.305	0.760		(−15.086, 11.032)	1.056
**Model 2**	(Constant)		62.651	7.480		8.376	< 0.001		(47.943, 77.359)	
	Patient education level	Primary school and below	Ref					0.424		
		Middle school	1.272	2.055	0.026	0.619	0.536		(−2.769, 5.312)	1.646
		High school level or above	2.867	2.193	0.063	1.308	0.192		(−1.444, 7.179)	2.247
	Underwent surgery	No	Ref					0.929		
		Yes	−0.145	1.617	−0.003	−0.090	0.929		(−3.324, 3.034)	1.220
	Treatment complications	No	Ref					0.001		
		Yes	5.397	1.622	0.115	3.326	0.001		(2.206, 8.587)	1.153
	Self-care ability	No dependence required	Ref					< 0.001		
		Mild dependence	6.702	1.742	0.149	3.847	< 0.001		(3.276, 10.127)	1.449
		Moderate dependence	3.489	2.492	0.053	1.400	0.162		(−1.412, 8.389)	1.368
		Severe dependence	−5.213	4.833	−0.037	−1.079	0.281		(−14.716, 4.290)	1.155
	Age (years)	≤ 44	Ref					0.001		
		45–59	−4.823	2.131	−0.105	−2.264	0.024		(−9.013, −0.634)	2.077
		≥ 60	−12.665	3.325	−0.214	−3.809	< 0.001		(−19.202, −6.127)	3.037
	Family member education level	Primary school and below	Ref					< 0.001		
		Middle school	3.932	2.499	0.067	1.573	0.117		(−0.982, 8.845)	1.735
		Junior college	8.326	2.722	0.150	3.059	0.002		(2.975, 13.677)	2.291
		Bachelor's degree or above	13.397	2.942	0.266	4.554	< 0.001		(7.613, 19.181)	3.266
	Employment status	Unemployed	Ref					0.767		
		Farmer	−2.056	4.885	−0.015	−0.421	0.674		(−11.660, 7.549)	1.284
		Retired	2.878	3.084	0.051	0.933	0.351		(−3.185, 8.941)	2.862
		Employed	1.036	2.496	0.023	0.415	0.678		(−3.873, 5.945)	2.899
	Religious belief	No	Ref					0.052		
		Yes	−7.862	4.037	−0.068	−1.948	0.052		(−15.800, 0.075)	1.158
	Family per capita monthly income (CNY)	≤ 2000	Ref					< 0.001		
		2001–5000	3.795	3.015	0.082	1.259	0.209		(−2.132, 9.723)	4.038
		> 5000	12.290	3.597	0.274	3.416	0.001		(5.217, 19.364)	6.187
	First-degree relatives of the patient	No	Ref					0.072		
		Yes	4.380	2.424	0.098	1.807	0.072		(−0.385, 9.145)	2.799
	Situation of living with patient	Not living with the patient	Ref					0.653		
		Living with the patient all the time	1.819	2.366	0.040	0.769	0.442		(−2.833, 6.471)	2.653
		Living intermittently with the patient	1.880	2.614	0.027	0.719	0.473		(−3.260, 7.020)	1.315
	Place of residence	Rural area	Ref					0.829		
		Suburb area	1.421	2.329	0.027	0.610	0.542		(−3.159, 6.001)	1.861
		Urban area	0.736	2.145	0.016	0.343	0.732		(−3.482, 4.954)	2.160
	Medical insurance	Basic medical insurance	Ref					0.648		
		Supplementary insurance	0.428	1.924	0.008	0.222	0.824		(−3.356, 4.211)	1.224
		Self-funded	6.342	6.920	0.031	0.916	0.360		(−7.264, 19.948)	1.093
	Communicating with the doctor about patient's condition	No	Ref					0.265		
		Yes	2.428	2.174	0.041	1.117	0.265		(−1.846, 6.703)	1.298
	Other relatives who had cancer	No	Ref					0.972		
		Yes	−0.131	3.686	−0.002	−0.035	0.972		(−7.378, 7.117)	4.202
	Participating in the care of other relatives with cancer	No	Ref					0.001		
		Yes	11.997	3.668	0.125	3.270	0.001		(4.784, 19.210)	1.405
	Experienced cancer bereavement	No	Ref					0.822		
		Yes	0.858	3.811	0.014	0.225	0.822		(−6.636, 8.352)	3.813
	Diagnosis of related diseases	No	Ref					0.628		
		Yes	0.917	1.890	0.019	0.485	0.628		(−2.799, 4.632)	1.406
	Self-rated health	Poor	Ref					< 0.001		
		Average	−3.339	2.803	−0.074	−1.191	0.234		(−8.850, 2.172)	3.730
		Good	−13.322	3.136	−0.295	−4.248	< 0.001		(−19.488, −7.156)	4.633
	Change in health status	Worse	Ref					< 0.001		
		No change	−3.457	2.111	−0.069	−1.637	0.102		(−7.609, 0.694)	1.682
		Improved	−15.578	3.839	−0.159	−4.058	< 0.001		(−23.127, −8.029)	1.479

The “*P*” values refer to tests of individual dummy-coded categories compared with the reference category. The “Overall P” values are from partial *F* tests, and refer to the overall tests for each multilevel categorical factor.

Model 1: *F* (7, 406) = 8.285, *P* < 0.001, *R*^*2*^ = 0.125, adjusted *R*^*2*^ = 0.110, Δ*R^2^* = 0.125.

Model 2: *F* (34, 379) = 17.068, *P* < 0.001, Δ*F* (27, 379) = 17.052, *R*^*2*^ = 0.605, adjusted *R*^*2*^ = 0.569, Δ*R^2^* = 0.480.

The Durbin–Watson statistic was 1.790. Residual plots suggested that the assumptions of linearity, homoscedasticity, and approximate normality of residuals were acceptable. Standardized residuals ranged from −3.088 to 3.076, and two observations had an absolute value slightly greater than 3. Further inspection of these observations did not identify any data entry errors, measurement errors, or violations of the inclusion criteria. Leverage values ranged from 0.030 to 0.266. Further assessment of Cook's distance showed a maximum value of 0.038, indicating that these observations did not exert a disproportionate influence on the model estimates. Therefore, all valid observations were retained in the final analysis.

## Discussion

### Key results and interpretation

This study assessed cancer risk perception among family members of patients with digestive tract cancers and identified associated factors from both patient and family member perspectives. The findings offer evidence to guide health care providers in implementing targeted cancer prevention education and facilitating early screening among family members who may have elevated risk.

This survey revealed a total cancer risk perception score of 89.23 ± 22.42 among family members, with a mean item score of 3.43 ± 0.86. Across the four dimensions, affective risk perception had the highest mean item score (3.89 ± 1.49), whereas carcinogenic factor risk perception had the lowest mean item score (2.86 ± 0.77). This relative pattern warrants further study. In addition to general public anxiety about cancer and close familial relationships, family members often face long-term caregiving pressure during cancer diagnosis, treatment, and rehabilitation, which may increase their psychological burden and sensitivity to cancer-related risks.^24^ Inadequate access to accurate disease information and intuitive concerns about familial clustering or hereditary risk may strengthen affective risk perception. Therefore, nursing interventions should not only provide risk information, but also help family members interpret their emotional concerns appropriately and develop accurate, appropriately calibrated understanding of digestive tract cancer risk.

Empirical studies on risk perception among family members for digestive tract cancers remain limited, particularly those using multidimensional scales. Some studies have found that the risk perception among first-degree relatives of patients with gastric or colorectal cancer is relatively low or uncertain.^13^^,^^15^ However, others found higher perceived risk among family members of patients with cancer.^14^ These discrepancies may be attributed to differences in cancer type, measurement tools, cultural context, family communication, health literacy, and access to screening or genetic counseling services. Moreover, single-item measures mainly capture perceived probability, whereas multidimensional scales assess susceptibility, severity, worry, and controllability, which may further explain differences across studies. To address these gaps, future research should use more standardized multidimensional tools, and develop culturally tailored risk communication, health education, and risk prediction models for family members of patients with digestive tract cancer.^25^

Cancer risk perception was associated with both patient-related and family member-related factors. From a nursing perspective, family members are influenced by the patient, but they may also influence cancer risk perceptions through resource access, health cognition, and caregiving exposure. Socioeconomic status affects access to health information, screening services, professional consultation, and social support; limited resources may increase uncertainty and reduce perceived control. Health cognition determines how family members interpret the patient's diagnosis, and insufficient cancer knowledge or health literacy may lead them to overestimate their own susceptibility. Caregiving experience exposes them repeatedly to symptoms, treatment side effects, recurrence concerns, and prognostic uncertainty, thereby strengthening emotional risk perception. Therefore, oncology nurses should regard family members not only as care providers but also as individuals with information needs and emotional vulnerability.

On the patient side, treatment complications and mild dependence in self-care ability were identified as associated factors of cancer risk perception among family members. The presence of treatment complications was positively associated with higher risk perception. This may be because complications make the disease and treatment burden more visible to family members, thereby intensifying their awareness of cancer severity. In addition, mild dependence in self-care ability was associated with higher family members' risk perception compared with no dependence. Impaired self-care ability may increase family members' caregiving involvement and exposure to the patient's illness trajectory, thereby strengthening perceived vulnerability.

Regarding family members' own characteristics, the final model showed that age 45–59 years and ≥ 60 years, junior college and bachelor's degree or above, family per capita monthly income > 5000 CNY, participation in caring for other relatives with cancer, good self-rated health, and improved health status were associated with cancer risk perception. Compared with family members aged ≤ 44 years, those aged 45–59 years and ≥ 60 years had lower cancer risk perception scores, suggesting that younger family members may be more sensitive to cancer-related information or more concerned about future health risks. Family members with junior college and bachelor's degree or above had higher risk perception scores, indicating that higher educational attainment may enhance access and interpretation of cancer-related information. Family members with family per capita monthly income > 5000 CNY also had higher risk perception scores than those with income ≤ 2000 CNY. This might be because people with higher incomes are more likely to obtain health information, screening resources, and professional consultations. These findings align with multiple studies reporting positive correlations between income/education and risk perception, and an inverse relationship with age.^26^^,^^27^ A divergent finding was reported in a study of colorectal cancer risk perception among African American men, which indicated that older age was linked to higher risk perception.^28^ This might be related to cultural background and the different survey population, and the underlying mechanisms need to be further explored.

Family members with prior caregiving experience for other people with cancer demonstrated heightened perception of cancer risk. Direct exposure to cancer diagnosis, treatment, and caregiving processes may increase their awareness of cancer susceptibility and prevention. A previous study found that a cancer diagnosis in family influences an individual's health-related belief and risk perception, which can increase the likelihood of cancer screening.^29^ This indicates that the experience of caregiving may make cancer more personally relevant and enhance their perception of the cancer risk. Family members with good self-rated health had lower cancer risk perception scores than those reporting poor health. Similarly, those whose health status had improved had lower risk perception scores than those whose health status had worsened. These findings suggest that perceived poor health or health deterioration may increase bodily vigilance and perceived susceptibility, especially when combined with cancer-related worry and insufficient knowledge. Thus, caregiver support should include assessment of both physical health and cancer-related worry.

The findings may be understood in the context of family communication and shared illness experience. Cancer diagnosis and treatment often influence not only patients themselves but also their family members, who may acquire disease-related information through daily communication, caregiving involvement, and observation of the patient's illness trajectory. Family members who are more involved in caregiving may become more aware of cancer-related risks and may interpret the patient's illness experience as personally relevant. In addition, emotional responses such as fear, uncertainty, or perceived vulnerability may be transmitted within families, shaping family members' perception of their own cancer risk.^30^ However, as the present study did not conduct formal dyad-level analyses, these explanations should be interpreted as potential mechanisms rather than direct evidence of mutual influence between patients and family members.

### Implications for practice

Based on the present findings, oncology nurses should provide staged and targeted interventions for family members of patients with digestive tract cancer. During hospitalization, nurses may identify family members with potentially inaccurate or poorly calibrated risk perceptions by briefly assessing family history, cancer-related knowledge, perceived susceptibility, and emotional response, and then provide bedside education using plain language, visual materials, and teach-back methods. For older family members or those with lower educational levels, information should be simplified, repeated in short sessions, and linked to concrete risk factors such as family history, *Helicobacter pylori* infection, dietary habits, and precancerous lesions. During outpatient consultations, nurses can further provide individualized risk communication based on patients' diagnosis, family history, and family members’ health literacy. Family members who meet relevant guideline-based eligibility criteria may be referred to genetic counseling or appropriate screening services when needed. For family members with lower income, nurses can recommend available resources to them, such as free screening programs, information on insurance coverage and community health services. During continuous care after discharge, telephone follow-up, WeChat-based education, and community linkage may be used to reinforce risk information, correct misconceptions, and support sustained preventive behaviors. In particular, family members of patients with treatment complications or impaired self-care ability should be guided to pay attention to their own health while caring for the patient. Therefore, oncology nursing interventions should shift from general health education to scenario-based, subgroup-specific risk communication integrated with inpatient care, outpatient counseling, and long-term follow-up. The clinical goal should be accurate and appropriately calibrated risk understanding, rather than simply increasing the total risk-perception score.

### Limitations

This study was conducted only in Chengdu, Sichuan Province, and the participants were recruited using consecutive sampling. The sample may not fully represent all patients with cancer and their family members. Future multi-center studies need to adopt probability-based sampling and larger, more diverse samples to enhance representativeness. Additionally, cross-sectional studies can only indicate the correlations between variables rather than establish causal inferences. The variables included in this study were relatively limited. Although the regression model explained more than 50% of the variance, suggesting that the current variables have certain explanatory and practical value, several theory-driven factors, such as health beliefs, health literacy, social support, coping styles, and screening behaviors, were not assessed. Future studies should incorporate these psychosocial and behavioral variables within established theoretical frameworks to further improve model explanatory power. The revised scale in this study only underwent exploratory factor analysis, and the confirmatory factor analysis was not conducted. We plan to increase the sample size in our subsequent research to further verify the structural validity of the scale. Furthermore, although patients and family members were recruited in pairs, cancer risk perception was assessed only among family members. Formal dyad-level analyses could not be conducted. In addition, there may also be response biases caused by the mixed questionnaire management models.

## Conclusions

Cancer risk perception score of family members of patients with digestive tract cancer was 89.23 ± 22.42. Among the four dimensions, carcinogenic factor risk perception had the lowest mean item score. Cancer risk perception among family members was associated with both patient-related and family member-related factors. These findings support the need for targeted risk communication to promote accurate understanding of digestive tract cancer risk and informed preventive decision-making.

## CRediT authorship contribution statement

Luling Zhou: Conceptualization, Methodology, Software, Investigation, Data curation, Writing – original draft. Lingzhi Yang, Lang Wang: Data curation. Jiao Zhou: Writing – review and editing. Ka Li: Supervision. All authors have read and approved the final manuscript.

## Ethics statement

This study was approved by the Biomedical Ethics Committee of West China Hospital, Sichuan University (Approval No. 2022–1261) and was conducted in accordance with the Declaration of Helsinki and its subsequent amendment. All participants provided informed consent.

## Data availability

The datasets used and analyzed during this study are available from the corresponding author upon reasonable request.

## Declaration of generative AI and AI-assisted technologies in the writing process

No AI services were used during the preparation of this work.

## Funding

This study was supported by the Key R&D project of Sichuan Provincial Department of Science and Technology, China (Grant No. 2024YFFK0034). The funders had no role in the study design; data collection, analysis, or interpretation; preparation of the manuscript; or the decision to submit the article for publication.

## Declaration of competing interest

The authors declare no conflict of interest.
